# Retraction: The Different Role of Notch1 and Notch2 in Astrocytic Gliomas

**DOI:** 10.1371/journal.pone.0224117

**Published:** 2019-10-15

**Authors:** The PLOS ONE Editors

Concerns have been raised about several results reported in this article [1]:

- The second and last FACS plots in Fig 2C (U251/Empty Vector and A172/Notch2) appear similar, although the listed percentages are different.
- In Fig 3, U251 NF-κB panel lanes 1, 2 appear similar to A172 PCNA panel lanes 2, 1, respectively, when flipped vertically and horizontally. Additionally, the U251 MMP2 panel appears similar to the A172 PI3K panel, the U251 Cyclin D1 panel appears similar to the A172 Bcl-2 panel, and there are similarities between the U251 Caspase 3 panel and the A172 Notch2 panel.
- In Fig 4, several mice appear to be included in multiple panels as representing different experimental results. The authors reported that errors were made in generating Fig 4 such that images from control experiments were used as representing Scramble Notch1 siRNA (Scr siRNA) and Empty Vector results, and images from the Scramble Notch1 siRNA (Scr siRNA) group were used as representing Notch2 results.
- The tumor sizes reported in Fig 4 exceed sizes commonly accepted for mouse tumor studies. In response to journal queries, the authors did not provide scientific or ethical justification for the tumor sizes, and they clarified that no specific criteria (i.e., humane endpoints) were used to determine when animals were euthanized. They explained that they typically terminated the experiment after 3–4 weeks, when quality of life was “seriously affected” in the group bearing the largest tumors.
- In Fig 6, there is overlap between the lower half of the Notch1 siRNA and the upper half of the Notch2 plasmid images. The authors commented that this resulted from a figure preparation error and that the images are both from the Notch2 siRNA experiment.
- In the published Fig 6, there are areas where the TUNEL staining pattern (green) appears similar although the DAPI staining (blue) differs, when comparing the Control, Scramble Notch1 siRNA (Scr siRNA), and Empty Vector panels; and when comparing the Notch1 siRNA and Notch2 plasmid panels. There are also areas in the Control and Scr siRNA panels where DAPI staining patterns appear similar, although regions adjacent to these areas differ.

The authors offered updated versions of Figs 4 and 6 in which the panels in question were replaced to address image duplications, but they noted that the original data supporting results reported in this article are no longer available.

The *PLOS ONE* Editors retract this article in light of the above issues which call into question the validity and reliability of the reported results and raise concerns about animal welfare considerations in the study design.

AZ agreed with the retraction. The other authors did not respond or could not be reached.
